# Neurofilament-light chains (NF-L), a biomarker of neuronal damage, is increased in patients with severe sarcopenia: results of the SarcoPhAge study

**DOI:** 10.1007/s40520-023-02521-9

**Published:** 2023-08-15

**Authors:** Aurélie Ladang, Stéphanie Kovacs, Laetitia Lengelé, Médéa Locquet, Charlotte Beaudart, Jean-Yves Reginster, Olivier Bruyère, Etienne Cavalier

**Affiliations:** 1 Clinical Chemistry Department, CHU de Liège, University of Liège, Avenue de L’Hopital, 1, 4000 Liège, Belgium; 2 WHO Collaborating Centre for Public Health Aspects of Musculoskeletal Health and Aging, Division of Public Health, Epidemiology and Health Economics, University of Liège, 4000 Liège, Belgium; 3 Physical, Rehabilitation Medicine and Sports Traumatology, SportS2, CHU de Liège, University of Liège, 4000 Liège, Belgium

**Keywords:** Sarcopenia, Biomarkers, Neurofilaments, NfL

## Abstract

**Background:**

As clinical tests, such as gait speed, require nervous system integrity to be performed properly, sarcopenia shares features with neurological diseases. Neurofilament light chains (NF-L) are now used as a blood-biomarker of neuronal damage, and its expression might be altered in sarcopenia. We aimed to assess NF-L concentrations in a large cohort of older individuals screened for sarcopenia.

**Methods:**

The SarcoPhAge cohort is a Belgian cohort of 534 community-dwelling older adults with an ongoing 10-year follow-up. Sarcopenia diagnosis was established at inclusion according to the European Working Group on Sarcopenia in Older People 2 (EWGSOP2) criteria. Muscle strength was evaluated with a hydraulic hand dynamometer, appendicular lean mass by Dual-Energy X-ray Absorptiometry (DXA) and physical performance by the Short Physical Performance Battery (SPPB). NF-L was measured on all available sera collected at the time of inclusion (*n* = 409) using SiMoA technology (Quanterix°).

**Results:**

In the multivariate model, NF-L was associated with performance tests such as gait speed (*p* < 0.0001) and SPPB scores (*p* = 0.0004). An association was also observed with muscle strength (*p* = 0.0123) and lean mass (*p* = 0.0279). In the logistic regression model, NF-L was an independent predictor of severe sarcopenia (*p* = 0.0338; OR = 20.0; 95% CI 1.39–287.7) with satisfactory diagnostic accuracy (AUC: 0.828) and subjects with an SPPB score ≤ 8 had higher odds of having increased NF-L (*p* < 0.0001; OR = 23.9; 95% CI 5.5–104).

**Conclusions:**

These data highlight the potential for using NF-L to investigate the pathophysiology of sarcopenia severity and the neurological features associated with performance tests. However, these results need to be confirmed with other cohorts in different settings.

**Supplementary Information:**

The online version contains supplementary material available at 10.1007/s40520-023-02521-9.

## Introduction

Sarcopenia is a musculoskeletal disease that lowers quality of life and is associated with an increased risk of falls and fractures, higher mobility disorders and loss of independence [1–4]. Sarcopenia diagnosis is based on three determinants: the loss of muscle strength, reduced muscle mass and decreased physical capacity [5]. Several definitions of the disease coexist and according to the chosen definition, the overall contribution of these three determinants to the diagnosis of sarcopenia is different [3, 5–7]. According to the EWGSOP2 definition, the loss of muscle strength is the major determinant of sarcopenia since the prognostic value on negative outcomes and particularly on all-cause mortality is high [8]. Muscle strength is usually evaluated by measuring grip strength with a hand dynamometer to evaluate the presence of probable sarcopenia [5]. The evaluation of the muscle mass should be used in a second time to confirm the sarcopenia diagnosis after screening for low muscle strength [5]. Indeed, measurement of muscle mass is hardly accurate and requires more expensive imaging techniques such as Dual-energy X-ray absorptiometry (DXA) [9]. Finally, physical performance tests are performed to evaluate the severity of the disease. These tests usually involve gait speed, balance and timed-up-and-go measurements [5]. A physical performance test combines the evaluation of the chair stand, the gait speed and the balance tests. This test is called the short physical performance battery (SPPB) test and is graded from 0 to 12 points, 0 being the worst-case scenario [10]. However, in the AWGS definition, gait speed is not a severity criterion but rather a direct criterion for sarcopenia [6].

In parallel to this definition, several reviews have argued that sarcopenia is not only a muscle disease but that it also shares features with neurological disorders [11–14]. Indeed, many age-related physiological and pathological changes in innervation deteriorate muscle force and physical performance [15]. These changes include instability of the neuromuscular junction or alteration in myofiber calcium homeostasis [12]. Additionally, some studies have reported an association between sarcopenia and cognition [16, 17]. Associations between cognition or delirium and handgrip strength, muscle mass or gait speed are also well characterized [18–20]. Furthermore, two sarcopenia diagnosis criteria, namely, grip strength and performance tests, are based on volitional tasks that require the integrity of the nervous system to be properly executed [13]. Physical performance tests are even defined by the EWGSOP2 committee as “a multidimensional concept that not only involves muscles but also central and peripheral nervous function, including balance” [5].

In a recent ESCEO consensus paper, the measurement of biomarkers of the neuromuscular junction was recommended to be assessed in clinical trials of drugs aiming at sarcopenia management [21]. However, so far, only the C-terminal fragment of agrin (CAF) and brain-derived neurotrophic factor have been addressed to this end with huge analytical limitations for CAF, leaving space for the evaluation of other neurological biomarkers. This consensus paper also points to neurofilament light chains (NF-L) as a promising biomarker for which more studies are needed. NF-L is an axonal protein that has been suggested to serve as a panbiomarker of nervous system alteration, and its concentration is modified in many conditions affecting either the central or peripheral system [22]. NF-L specificity to neurons even enables a distinction between neurological and psychiatric disorders [23]. In a previous study on the same cohort, we identified that in addition to neurological function and cognition, major confounding factors influencing NF-L expression are renal function, BMI, and age [24].

Regarding sarcopenia, NF-L has been associated with the skeletal muscle index (SMI) and muscle strength in a cohort of middle-aged and older adults [25], but NF-L has not been evaluated together with the three determinants of sarcopenia in a single study. In this context, this study aimed to better decipher the links between major sarcopenia criteria and neuronal damage by evaluating the contribution of NF-L to each determinant of sarcopenia as well as the ability of NF-L to diagnose sarcopenia or sarcopenia severity according to the EWGSOP2 criteria.

## Methods

### Sample collection

All subjects participated in a long-term prospective study called the SarcoPhAge study (for sarcopenia and physical impairment with advancing age). This study follows 534 individuals aged between 65 and 92 years at inclusion and with an ongoing follow-up after 10 years to evaluate the quality of life and the consequences of sarcopenia in older community-dwelling Belgian subjects. The only exclusion criteria were limb amputation or body mass index (BMI) above 50 kg/m^2^. The complete methodology has been described elsewhere [26]. All patients gave written and signed informed consent and the study was approved by the Ethical Committee of the University Teaching Hospital of Liège (2012/277).

Among these 534 individuals, 409 participants had a serum sample collected at inclusion time. All 409 samples were assessed for NF-L.

### Diagnosis of sarcopenia

The diagnosis of sarcopenia was based on the EWGSOP2 criteria as defined in Cruz-Jentoft et al*.* [5]. Appendicular lean mass (ALM) was measured by daily calibrated dual-energy X-ray absorptiometry (DXA) to determine lean mass. The skeletal muscle mass index (SMI) was calculated by adding the skeletal lean mass of both arms and legs and normalized by dividing by height squared (ASM/height^2^). A hydraulic dynamometer (Saehan Corporation, MSD Europe Bvba, Belgium) was used to evaluate handgrip strength (HGS). Three measurements on each arm (six measurements in total) per individual were recorded and muscle strength was defined as the highest of the six measurements [27]. Criteria for sarcopenia were defined as handgrip strength below 27 kg for men and 16 kg for women combined with ASM/height^2^ below 7.0 kg/m^2^ for men and 5.5 kg/m^2^ for women.

According to the EWGSOP2 definition, low muscle strength and low lean mass are mandatory to diagnose sarcopenia and physical performance only indicates the severity of the disease. Physical performance was assessed through the Short Physical Performance Battery (SPPB). SPPB test is composed of 3 different tests (balance, 4-m gait speed and the chair stand test), each scored 0–4 points for a maximum of 12 points. Among people with sarcopenia, an SPPB score below 8 was used to classify subjects as having severe sarcopenia.

### NF-L and cystatin C measurements

Serum NF-L was analyzed with SiMoA° technology on an SR-X platform (Quanterix, USA) following the manufacturer’s instructions (for NF-L assay performance specifications, see Ladang et al*.* [24]). Serum-standardized cystatin C was measured with an immunoturbidimetric assay (Tina-quant Cystatin C Gen.2 assay) on Cobas C6000 (Roche, Germany). Estimated glomerular filtration rate (eGFR) was calculated using the CKD-EPI equation for cystatin C only [28].

### Statistical analysis

First, data were tested for the normality of the distribution for all continuous variables using the Shapiro–Wilk test. Continuous variables not normally distributed are expressed as medians and 25th–75th percentiles. Spearman’s correlation was used to assess the association between NF-L and sarcopenic determinants (HGS, ASM/height^2^, SPPB) in unadjusted models for continuous variables. When data were sorted by categories according to score, the Kruskall-Wallis test was used to assess significant differences between groups. Multiple linear regressions were performed with sarcopenic determinants as dependent variables and log NF-L, age, sex, number of drugs, number of concomitant diseases, Mini-Mental State Examination (MMSE) and body mass index (BMI) as co-variates. All the independent variables were entered into the model in one single step without checking for the level of significance. To check the association between NF-L and the diagnosis of sarcopenia and severe sarcopenia, a univariate analysis was performed using the Mann–Whitney test followed by a multivariate analysis with logistic regression with log NF-L, age, sex, number of drugs, number of concomitant diseases, MMSE and BMI as covariates. For the logistic regression model, all the independent variables were entered into the model, checked and removed when variables became non-significant. The level of statistical significance was set to 5%. All statistical analyses were performed using Medcalc (Medcalc software, Belgium).

## Results

### Cohort description

The demographic characteristics of the 409 individuals for which a serum was collected at the time of inclusion were previously described [24]. Briefly, the studied population was aged between 65 and 92 years old (median 72.4 years, percentile 25–75: 68.0—77.5 pg/mL) with 42.3% of males. A total of 5.6% of the individuals had a BMI below 20 and 21.8% had a BMI equal to or above 30. A total of 37.9% of the individuals had moderate-to-severe renal insufficiency (estimated glomerular filtration rate below 60). Thirty-six percent of the individuals were taking more than 7 drugs, and 7.6% had an MMSE ≤ 25. Approximately one-quarter of the individuals reported 2 or fewer comorbidities and another quarter reported more than 5 comorbidities (102 and 105 individuals, respectively). A total of 16 individuals (3.9%) were classified as sarcopenic patients according to the EWGSOP2 definition, among whom 12 were diagnosed with severe sarcopenia.

The median NF-L concentration of the whole cohort was 21.3 pg/mL (percentile 25–75: 15.6–29.2 pg/mL) and age, BMI, cystatin C concentration, reporting of neurological troubles and MMSE were found to be independent predictors of the NF-L concentration. The NF-L distribution was Gaussian after logarithmic transformation, and the mean log NF-L was 1.34 pg/mL (SD: 0.22).

### The NF-L concentration and the diagnostic criteria of sarcopenia

**Fig. 1:**
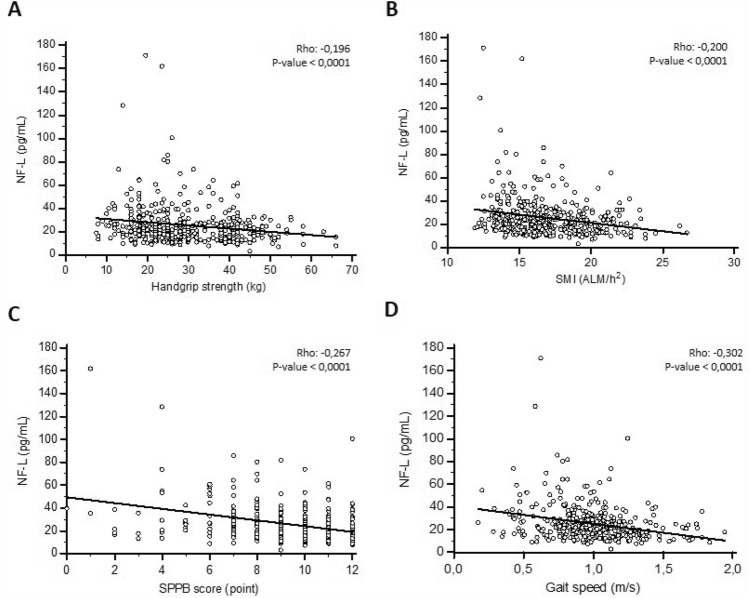
NF-L and sarcopenia determinants (**A**–**D**): Correlation and regression of NF-L with handgrip strength (**A**), SMI (**B**), SPPB (**C**) or gait speed (**D**). Continuous line represents the regression line

First, the NF-L concentration was correlated to each criterion of the sarcopenic definition. NF-L was increased in individuals with decreased muscle strength or decreasedlean mass. The rank correlation between the NF-L concentration and handgrip strength yielded a Spearman’s rho of − 0.196 (*p*-value < 0.0001), whereas for NF-L and SMI, Spearman’s rho was − 0.200 (*p*-value < 0.0001). NF-L was also increased with the severity criterion for sarcopenia evaluated by the decrease in SPPB score and the slowing-down of gait speed (Spearman’s rho: − 0.302 and − 0.267, respectively; both *p*-value < 0.0001) (Fig. 1a–d). The results were identical when log NF-L was assessed.

For the multivariate model, age, sex, increasing number of medications, increasing number of concomitant diseases, MMSE score and BMI were selected as confounding variables for sarcopenia according to Locquet et al*.* [29]. In a multiple regression adjusted for these potential variables, log NF-L was independently associated with handgrip strength (*R*_partial_ − 0.1247; *p*-value 0.0123) and SMI (*R*_partial_ − 0.1095; *p*-value 0.0279). To a stronger extent, log NF-L was also independently associated with the SPPB score (*R*_partial_ − 0.1747; *p*-value 0.0004) and gait speed (*R*_partial_ − 0.2011; *p*-value < 0.0001) (Table 1).

**Table 1:** Adjusted association between NF-L and sarcopenic determinants

Dependent variable	Handgrip strength		SMI		SPPB score		Gait Speed		Time up and go	
	*R*_partial_	*p*-value	*R*_partial_	*p*-value	*R*_partial_	*p*-value	*R*_partial_	*p*-value	*R*_partial_	*p*-value
Age (years)	− 0.2644	< 0.0001***	− 0.0095	0.8493	− 0.2425	< 0.0001***	− 0.1884	0.0001***	0.0992	0.0536
Sexe	− 0.7641	< 0.0001***	− 0.7359	< 0.0001***	− 0.2267	< 0.0001***	− 0.2152	< 0.0001***	0.1696	0.0009***
Number of drugs	− 0.1571	0.0016**	− 0.0762	0.1265	− 0.2012	< 0.0001***	− 0.1799	0.0003***	0.1663	0.0012**
Number of concomitant diseases	− 0.01358	0.7861	0.0357	0.4747	− 0.1687	0.0007***	− 0.1192	0.0169***	0.1093	0.033*
MMSE	0.07393	0.139	0.0815	0.1022	0.2071	< 0.0001***	− 0.1639	0.001***	− 0.1331	0.0095**
Body mass index (kg/m^2^)	0.04933	0.3238	0.6699	< 0.0001***	− 0.188	0.0001***	− 0.1946	0.001***	0.0709	0.1682
log NF-L (pg/mL)	− 0.1247	0.123*	− 0.1095	0.0279*	− 0.1747	0.0004***	− 0.2011	< 0.0001***	0.0097	0.8514

A second model was also generated with eGFR as an additional confounding variable, with eGFR being known to be a confounding variable of NF-L expression but not of sarcopenia determinants. In this model, log NF-L was no longer independently associated with handgrip strength (*R*_partial_ − 0.07865; *p*-value 0.1163) and SMI (*R*_partial_ − 0.0739; *p*-value 0.1397), whereas the associations with performance tests (SPPB score:*R*_partial_ − 0.1671; *p*-value 0.0008) and gait speed (*R*_partial_ − 0.2022; *p*-value < 0.0001) remained unchanged. eGFR was not independently associated with any of these four criteria (Supplementary Table S1).

### NF-L concentration and performance tests

**Fig. 2:**
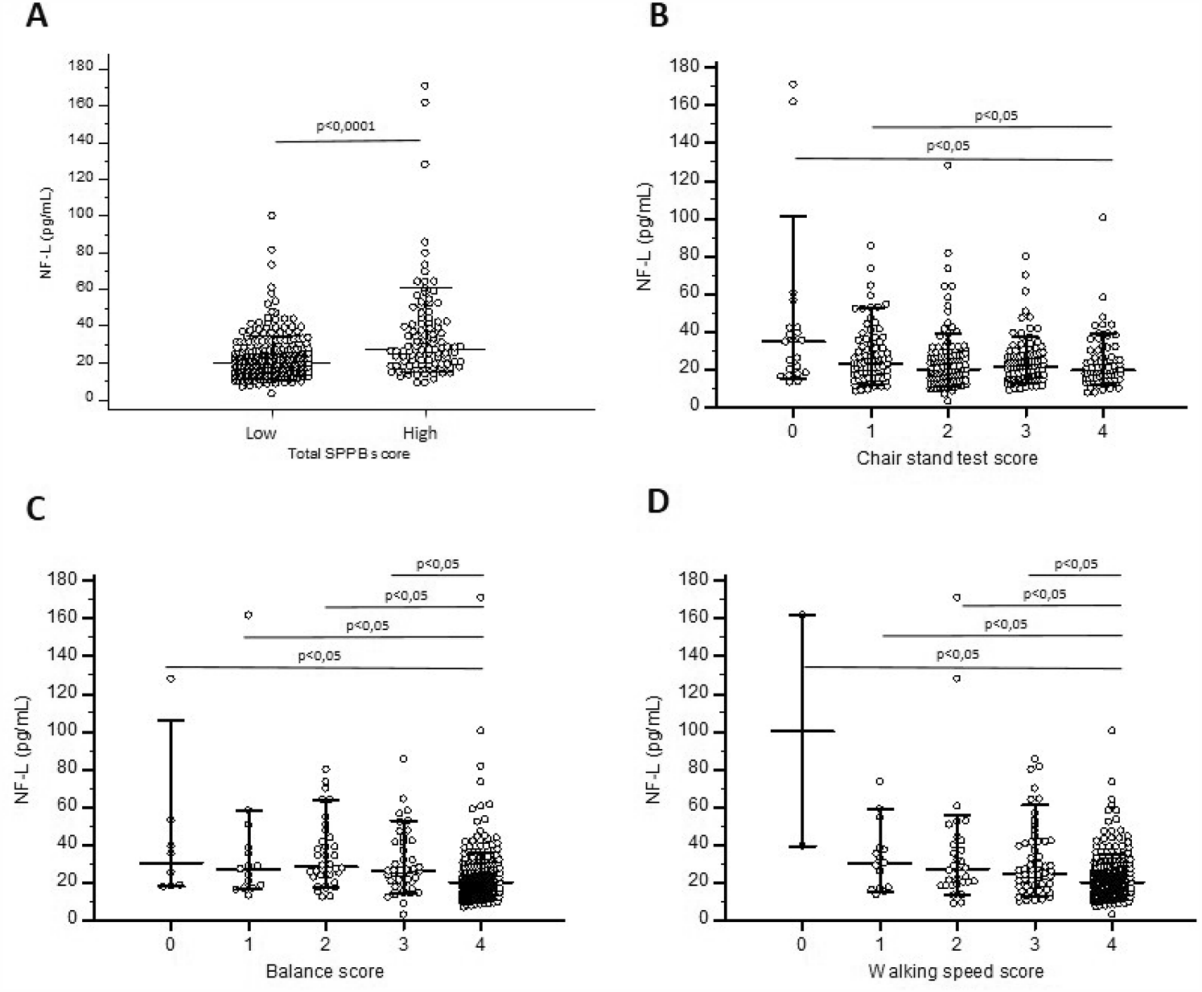
NF-L and performance tests (**A**–**D**): Scatterplot of NF-L of subjects classified according to their total SPPB score (**A**), Chair stand test score (**B**), Balance score (**C**) and Walking speed score (**D**). Medians as well as 10–90 percentiles are represented by horizontal lines, whereas each single result is represented by a dot

To better understand the association between SPPB score and NF-L, subjects were categorized into high (9–12 points) or low performance (0–8 points) according to SPPB tests and a logistic regression was performed. For each increase of 1 unit of NF-L, the risk of having a low SPPB score was increased of 23.9% (CI 5.5–104; *p* < 0.0001) (Fig. 2a). In the chair stand test score, subjects with a lower score (0–1 chair stand) had higher NF-L concentration compared to subjects with a full score of 4 (Kruskal–Wallis test: *p* < 0.0150) (Fig. 2b). In the balance score and in the walking speed score, subjects with the best score had lower NF-L concentration compared to all the other subgroups (Kruskal–Wallis test: *p* < 0.0001) (Fig. 2c, d).

Additionally, in multiple regression with the same potential confounding variables as above, an independent association between log NF-L concentration and balance score (*R*_partial_ − 0.1701; *p*-value 0.0006) was observed. This association was also observed when the walking speed score was assessed (*R*_partial_ − 0.1809; *p*-value 0.0003) but not with the chair stand test (*R*_partial_ − 0.0575; *p*-value 0.2493) (Table 2). When eGFR was added to our model, the same association was observed (supplementary Table S2). Finally, timed-up-and-go was not associated with log NF-L concentration in the multiple regression model with or without eGFR (Table 1 and Supplementary Table S1).

**Table 2:** Adjusted association between NF-L and performance tests

Dependent variable	Balance score		Walking speed score		Chair stand test score	
	*R*_partial_	*p*-value	*R*_partial_	*p*-value	*R*_partial_	*p*-value
Age (years)	− 0.2018	< 0.0001***	− 0.171	0.0006***	− 0.1443	0.0037**
Sexe	− 0.1169	0.0189*	− 0.1614	0.0012**	− 0.188	0.0001***
Number of drugs	− 0.0027	0.9573	− 0.2176	< 0.0001***	− 0.2064	< 0.0001***
Number of concomitant diseases	− 0.1311	0.0084**	− 0.1172	0.0186*	− 0.1202	0.0158*
MMSE	− 0.1947	0.0001***	− 0.1679	0.0007***	0.1093	0.0283*
Body mass index (kg/m^2^)	− 0.1229	0.0136*	− 0.1509	0.0024**	− 0.1398	0.0049**
log NF-L (pg/mL)	− 0.1701	0.0006***	− 0.1809	0.0003***	− 0.0575	0.2493

### The NF-L concentration and sarcopenic patients

**Fig. 3:**
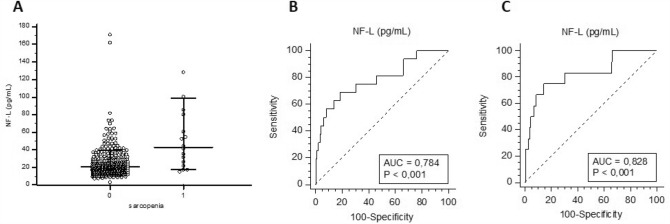
NF-L in sarcopenia: Scatterplot of NF-L of subjects with or without sarcopenia. Medians as well as 10–90 percentiles are represented by horizontal lines, whereas each single result is represented by a dot (**A**). ROC curve of NF-L for all sarcopenic subjects (**B**) and subjects with severe sarcopenia (**C**)

NF-L was increased in all sarcopenic patients (median NF-L: 43.0 pg/mL; percentile 25–75: 24.1–70.3) compared to controls (median NF-L: 21.1 pg/mL; percentile 25–75: 15.4–28.3) (Mann–Whitney; *p* = 0.0001) (Fig. 3a) as well as in severe sarcopenic patients (median NF-L: 48.4 pg/mL; percentile 25–75: 31.0–70.3) (Mann–Whitney; *p* = 0.0001). In a logistic regression model with the introduction of the same potential confounding variables as above, NF-L was found to be a predictor of severe sarcopenia. Indeed, the odds ratio for the log NF-L contribution to the model was 20.0 (95% CI 1.39–287.7). The results were unchanged by the addition of eGFR to the model. However, when considering all sarcopenic individuals, NF-L tended to be a predictor of the disease but did not reach the level of statistical significance (OR: 10.9; 95% CI 0.97–121.6) in models with and without eGFR.

When the diagnostic accuracy of NF-L was assessed as a biomarker of sarcopenia, the area under the curve (AUC) obtained through ROC analyses was 0.784 for all sarcopenic subjects (*p*-value < 0.001) with a sensitivity of 68.7% and a specificity of 81.4% for an optimal cut-off point at 31.6 pg/mL (Fig. 3b). For subjects with severe sarcopenia, the AUC was 0.828 (*p*-value < 0.001) with a sensitivity of 75.0% and a specificity of 85.6% for an optimal cut-off point at 35.5 pg/mL (Fig. 3c).

However, given that the NF-L concentration was shown to be tightly correlated with renal function and that renal function evaluated by cystatin was decreased in sarcopenic patients (Kruskal–Wallis test: *p*-value: 0.004), we checked whether the NF-L increase in sarcopenic subjects is associated with renal function. To do so, we decided to generate a multiple regression model where NF-L was defined as the dependent variable and the known confounding factors of NF-L concentration (age, MMSE, BMI and renal function according to Ladang et al. [24]) as well as sarcopenic diagnosis were defined as independent variables. In this model, log NF-L was independently associated with all sarcopenic subjects (*R*_partial_ 0.0986; *p*-value 0.0477) as well as with patients suffering from severe sarcopenia (*R*_partial_ 0.1022; *p*-value 0.0400) (Table 3).

**Table 3:** Adjusted model for NF-L and its covariates

Dependent variable: log NF-L (pg/mL)	*R*_partial_	*p*-value
Age (years)	0.2453	< 0.0001***
MMSE	− 0.0954	0.0553
Body mass index (kg/m^2^)	− 0.3031	< 0.0001***
Glomerular filtration rate	− 0.4344	< 0.0001***
All sarcopenic/severe sarcopenic	0.0986/0.1022	0.0477/0.0400*

## Discussion

In the present study, we assessed the measurement of NF-L as a biomarker of sarcopenia. Although NF-L was associated with muscle strength and lean mass, the association was stronger for performance tests such as gait speed or SPPB score. To the best of our knowledge, this study is the first to associate all sarcopenic determinants with NF-L in a single cohort. However, He et al. previously reported an association between NF-L and SPPB test, and between NF-L and handgrip strength in community-dwelling older adults without evaluating other sarcopenic determinants [30]. Additionally, Pratt et al. also showed that NF-L is associated with handgrip strength and SMI in community-dwelling older adults [25]. Compared to their results, we also observed a stronger association between muscle strength and NF-L compared to that between lean mass and NF-L [25]. However, we did not observe any contribution of sex to our multifactorial models. In fact, although several papers have reported that NF-L might have a different expression profile between the sexes, the current knowledge suggests that there is not a sex-mediated expression of NF-L in the general population, and this question has not been studied enough in sarcopenia to draw robust conclusions [31]. Of note, those two studies and ours were obtained with immunoassays using different technologies. Whereas He et al. used conventional ELISA, we and Pratt et al. used an ultrasensitive method from two different companies: Quanterix and Meso Scale Discovery (MSD), respectively. As there is no reference material for NF-L at the current moment, this can lead to differences in absolute values for NF-L between studies [32].

The stronger association between NF-L and performance tests is in line with the hypothesis that performance tests not only evaluate muscle integrity but also the ability of the central and peripheral nervous system to drive movement. Indeed, walking or chair standing are volitional tasks that require the integrity of the nervous system and that are affected by many neurological diseases [13]. If the hypothesis that the NF-L concentration reflects the neurological aspects of sarcopenia is true, then NF-L could help in distinguishing sarcopenia from other muscle-wasting syndromes, such as cachexia or frailty. Indeed, patho-physiological mechanisms of cachexia are related to a serious illness such as cancer and are not considered to share neurological features [33]. Regarding frailty, Lu et al. were unable to find an association between NF-L and frailty in older adults although the choice of the covariates has been commented on by other peers [34–36]. Therefore, assessing NF-L in other muscle wasting syndrome seems relevant, and NF-L might represent an interesting opportunity to increase the differential diagnostic accuracy of muscle wasting syndrome.

A second positioning of NF-L as a biomarker of sarcopenia could be the identification of sarcopenia severity or the identification of subjects with lower performance tests. Indeed, people with low SPPB tests had a higher chance of increased NF-L, and the associations between NF-L and handgrip strength or NF-L and SMI disappeared when eGFR was included in the multivariate model. Furthermore, according to the EWGSOP2 definition, performance tests are a not prerequisite for the diagnosis of the disease but rather a severity criteria [5]. The multivariate model for the association between NF-L and sarcopenia was only significant in the group suffering from severe sarcopenia. Additionally, diagnostic accuracy evaluated through AUC was better when only considering subjects suffering from severe sarcopenia. However, it is well known that sarcopenia prevalence depends on the definition that is used [3]. Therefore, NF-L might have better diagnostic accuracy for sarcopenia when criteria for the disease comprise those of performance tests, such as in AWGS criteria.

Nevertheless, several other considerations must be taken into account to define the positioning of NF-L as a biomarker of sarcopenia, and more studies are required before a clear statement can be made. First, it is necessary to confirm in other cohorts that the NF-L increase is independent of renal function. In fact, the NF-L increase observed both in sarcopenia and in severe sarcopenia in the univariate model was only confirmed for severe sarcopenia in our adjusted model. We also observed that our sarcopenic subjects had a reduced renal function, which might also drive the increase of NF-L concentration although both sarcopenia and renal function were independent predictors of NF-L concentration in a second adjusted model. Second, NF-L expression has been reported to be modified in a wide variety of neurological diseases [22, 31, 37]; thus, NF-L specificity for sarcopenia is low. Finally, many papers claim that sarcopenia being a multifactorial disease, the use of a single biomarker for diagnosis or follow-up of sarcopenia is unrealistic, and the use of a panel of biomarkers is the most relevant [38–40]. Therefore, the use of NF-L as a stand-alone diagnostic biomarker of sarcopenia is certainly not the most appropriate method but NF-L could be seen as a biomarker to investigate the neurological contribution to sarcopenia. Indeed, recently, pathophysiological mechanisms that should be investigated in clinical trials aiming at sarcopenia management have been defined, and these comprise the integrity of neuromuscular junction [21]. Since, NF-L has been widely addressed for the follow-up of drugs targeting multiple sclerosis with encouraging results [41], the use of NF-L, in combination with a panel of other muscle-specific biomarkers, might represent an interesting opportunity for the follow-up and the overall comprehension of neurological aspects linked to sarcopenia.

However, our study faces some limitations. First, given that our cohort comprises only 16 sarcopenic individuals (most of them with altered renal function), we could only conclude on a proper increase in NF-L in patients with severe sarcopenia. These results must therefore be confirmed in other cohorts with different settings. Second, because we only measured NF-L at a single time point, the use of NF-L for follow-up of the disease remains hypothetical and deserves further investigation with longitudinal data. Finally, more data are required to decipher the neurodegenerative part of the phenotype, as only MMSE was considered as a neurological covariate in our adjusted models.

In conclusion, NF-L appears to be linked to the severity of sarcopenia with a strong association with performance tests. Since not all muscle-wasting syndromes share neurological features, further research is needed to elucidate the role of neurological blood-based biomarkers such as NF-L in sarcopenia management.

### Supplementary Information

Below is the link to the electronic supplementary material.

Supplementary file 1 (DOCX 20 KB)
