# Supplementary material for: Neurofilament-light chains (NF-L), a biomarker of neuronal damage, is increased in patients with severe sarcopenia: results of the SarcoPhAge study
Source: Aging Clin Exp Res. 2023 Aug 15;35(10):2029–37. doi: 10.1007/s40520-023-02521-9 (PMC10520189; doi:10.1007/s40520-023-02521-9)
Supplement: Supplementary file 1 — (DOCX 20 KB) [file 40520_2023_2521_MOESM1_ESM.docx]

Supplementary table 1

Supplementary table 2 .
